# Development and validation of a nomogram for predicting new vertebral compression fractures after percutaneous kyphoplasty in postmenopausal patients

**DOI:** 10.1186/s13018-023-04400-5

**Published:** 2023-11-30

**Authors:** Jianhu Zheng, Yan Gao, Wenlong Yu, Ning Yu, Zetao Jia, Yanke Hao, Yungang Chen

**Affiliations:** 1 Affiliated Hospital of Shandong University of Traditional Chinese Medicine, No. 16369 Jingshi Road, Lixia District, Jinan, Shandong Province China; 2 Longhua Hospital, Shanghai University of Traditional Chinese Medicine, Shanghai, China; 3 Shandong University of Traditional Chinese Medicine, Jinan, China

**Keywords:** Postmenopause, Osteoporotic vertebral compression fractures, New vertebral compression fractures, Nomogram, Percutaneous kyphoplasty, Osteoporosis

## Abstract

**Background:**

Postmenopausal women face a heightened risk of developing new vertebral compression fractures (NVCFs) following percutaneous kyphoplasty (PKP) for osteoporotic vertebral compression fractures (OVCFs). This study aimed to develop and validate a visual nomogram model capable of accurately predicting NVCF occurrence post-PKP to optimize treatment strategies and minimize occurrence.

**Methods:**

This retrospective study included postmenopausal women diagnosed with OVCF who underwent PKP at the Affiliated Hospital of Shandong University of Traditional Chinese Medicine between January 2016 and January 2021. Patient data, including basic information, surgical details, imaging records, and laboratory findings, were collected. The patients were categorized into two groups based on NVCF occurrence within 2 years post-PKP: the NVCF group and the non-NVCF group. Following the utilization of least absolute shrinkage and selection operator (LASSO) regression for feature selection, a nomogram was constructed. Model differentiation, calibration, and clinical applicability were evaluated using receiver operating characteristic (ROC), calibration, and decision (DCA) curve analyses.

**Results:**

In total, 357 patients were included in the study. LASSO regression analysis indicated that cement leakage, poor cement diffusion, and endplate fracture were independent predictors of NVCF. The nomogram demonstrated excellent predictive accuracy and clinical applicability.

**Conclusions:**

This study used LASSO regression to identify three independent predictors of NVCF and developed a predictive model that could effectively predict NVCF occurrence in postmenopausal women. This simple prediction model can support medical decision-making and is feasible for clinical practice.

## Background

Osteoporosis (OP) is the most common systemic bone disease affecting human health and is characterized by trabecular microstructure degeneration, decreased bone mineral density, and compromised bone quality [1, 2]. It significantly increases the risk of spine and hip fractures [3], particularly in older females owing to the reduction in estrogen levels during perimenopause, which leads to increased receptor activator of NF-κB ligand expression and enhanced osteoclast activity. Consequently, osteoclasts become more active than osteoblasts, resulting in accelerated bone loss and increased susceptibility to fracture. On average, these patients experience an annual bone loss rate of approximately 2%, starting 1–3 years before menopause and persisting for 5–10 years [4, 5]. Fractures significantly restrict the daily activities of postmenopausal women, reduce their quality of life, and increase mortality rates [6].

Osteoporotic vertebral compression fractures (OVCFs), one of the most common complications of osteoporosis [7], can result from low-energy trauma, such as falls and severe coughing. Typically, OVCF causes severe lower back pain, particularly during positional changes, restricted movement, severe kyphosis, and potential lung function impairment [8]. In the USA, approximately 2 million osteoporotic fractures occur each year, with OVCF accounting for approximately 700,000 cases and predominantly affecting postmenopausal women [9]. In Europe, women aged > 50 years are twice as likely to experience vertebral compression fractures as men [10]. In China, the incidence of OVCF in women aged > 50 years is approximately 2.5 times higher than that in men [11].

Percutaneous kyphoplasty (PKP) is considered a safe and effective technique that js widely used to alleviate pain symptoms in the clinical treatment of OVCF [12, 13]. Nevertheless, studies have reported various postoperative complications, including cement leakage and new vertebral compression fractures (NVCFs) [14, 15], which significantly reduce the quality of life of patients and pose a substantial economic burden. In recent years, NVCF has drawn extensive attention from clinical practitioners.

Studies have reported that the incidence of NVCF following PKP surgery ranges from 6.5 to 34.8% [16, 17], with a 1-year follow-up study reporting an incidence of 19.2% in postmenopausal women [18]. The risk of developing NVCF increases after the first occurrence of OVCF and a history of fractures is considered an influential factor contributing to the development of new fractures in postmenopausal women [19, 20]. Numerous studies have focused on compression fractures and their associated risk factors, including bone cement diffusion, cement leakage, anti-osteoporosis treatments, and fracture location [16, 21, 22]. High-risk factors associated with NVCF include paraspinal muscle degeneration, bone cement leakage into the intervertebral disc, initial fracture occurring at the thoracolumbar junction, natural progression of osteoporosis, and the injected volume of bone cement [23–25]. Moreover, a risk factor analysis conducted in 2021 revealed that the proportion of women in the refracture group after OVCF (83.1%) was significantly higher than that of the non-fracture group (72.4%), establishing female sex as an independent risk factor for postoperative NVCF [26].

Nomograms are visual mathematical models used to predict the incidence of clinical events through complex operations [27], with wide applications in predicting patient prognosis. Nomograms allow clinicians to evaluate the prognostic risk of patients more clearly and formulate follow-up treatment plans, with significant value in guiding clinical treatment [28]. Given the substantial physical, psychological, and social burdens associated with NVCF, this study aimed to explore the independent predictors of NVCF in postmenopausal women after PKP by employing least absolute shrinkage and selection operator (LASSO) regression to establish a nomogram capable of predicting postoperative NVCF to support clinical decision-making.

## Methods

### Clinical data and selection criteria

This retrospective study analyzed 357 postmenopausal women who were diagnosed with osteoporotic compression fractures and subsequently treated with PKP at the Affiliated Hospital of Shandong University of Traditional Chinese Medicine between January 2016 and January 2021. All patients were followed up for 2 years. The study protocol was approved by the Research Ethics Committee of the Affiliated Hospital of Shandong University of Traditional Chinese Medicine. The surgical process of PKP was carefully explained to the patient before surgery and written informed consent was obtained from all patients.

The inclusion criteria were as follows: (1) postmenopausal women with primary osteoporosis; (2) availability of complete preoperative basic data, imaging data, and laboratory findings, with reexamination at a specified time post-PKP; (3) the presence of significant lower back pain (visual analog scale > 6) [21] and limited physical activity, particularly when turning over or getting up; (4) magnetic resonance imaging (MRI) showing distinct signal changes in the thoracolumbar bone, hyperintense T2 signals, and hypointense T1 signals in the fractured vertebrae, or whole-body bone scans indicating active bone metabolism; (5) an intact posterior wall of the vertebral body, with no involvement in the fracture or spinal canal invasion; and (6) the absence of preoperative organ failure.

The exclusion criteria were as follows: (1) OVCF caused by a tumor, infection, or tuberculosis; (2) systemic disease-related coagulation dysfunction that rendered the patient unable to tolerate surgery; (3) the presence of preoperative systemic or local infection; (4) evidence of spinal cord compression and prominent neurological symptoms, such as lower limb numbness or muscle atrophy; (5) previous posterior pedicle screw fixation and bone graft fusion; and (6) mechanical instability caused by rupture of the posterior wall of the vertebral body.

### Percutaneous kyphoplasty

All patients underwent PKP using a unilateral pedicle approach, conducted independently by experienced spine surgeons. The patients were placed in a prone position with support cushions under the chest and pelvis to prevent abdominal compression. Standard preoperative draping and disinfection procedures were followed, with continuous monitoring of vital signs throughout. C-arm fluoroscopy was performed for localization prior to needle insertion. In the anteroposterior view, the projection lengths of the spinous processes to both pedicles were aligned to appear as a single straight line, with the upper and lower endplates merging into a single continuous line. In the lateral view, the alignment of the anterior and posterior walls and pedicular margins formed a straight line, indicating successful fluoroscopy. A puncture site, located 3–4 cm lateral to the spinous process, was selected. Initially, a 4.2 mm diameter three-blade puncture needle (KZ001; Zhejiang Kehui Medical Equipment Co., Ltd., Zhejiang, China) was inserted at an angle of 30°–45° along the pedicle, entering the posterior third of the vertebral body. Subsequently, the stylet was removed and a solid drill (QJ902; Zhejiang Kehui Medical Equipment Co., Ltd., Zhejiang, China) was advanced through the puncture channel into the anterior third of the vertebral body under fluoroscopic guidance. In the anteroposterior view, active bleeding was observed when the solid drill reached the spinous process. Next, a 3.3 mm diameter balloon (QJ902; Zhejiang Kehui Medical Equipment Co., Ltd., Zhejiang, China) was inserted along the channel, gradually inflated, and maintained at pressure to expand the vertebral body and partially restore vertebral height. The balloon was removed under fluoroscopy, and bone cement material (OSTEOPAL V; Heraeus Medical GmbH, Wehrheim, Germany), mixed and kneaded to a toothpaste-like consistency, was slowly injected into the vertebral body using a cement injector (QJ902; Zhejiang Kehui Medical Equipment Co., Ltd., Zhejiang, China). The stylet was then reinserted into the channel, and the patients were advised to perform gentle lower limb movements for 2–5 min to ensure proper cement distribution. Once the cement had hardened, the puncture needle was withdrawn slowly. Finally, sterile dressings were applied over the incision and pressure was maintained for 3 min to complete the surgical procedure.

### New OVCF identification criteria

The main diagnostic criteria for identifying NVCF after PKP were: recurrent lower back pain accompanied by limited movement, such as difficulties in getting up and turning over, within 2 years of follow-up after the initial PKP-treated OVCF; physical examination revealing local tenderness and percussion pain, without evidence of spinal cord compression or nerve root symptoms; and comparison of radiographic findings between the first OVCF and subsequent assessments revealing the presence of a new vertebral wedge, with MRI showing a low signal intensity on T1-weighted images and a high signal intensity on T2-weighted images. MRI was also used to rule out other spinal conditions, including infections and malignant tumors, thereby assisting in the exclusion of patients who did not meet the NVCF criteria (i.e., the non-NCVF group). Patients who met these criteria were included in the NVCF group.

### Selection criteria for predictor variables

An extensive and comprehensive literature review was conducted to identify the risk factors associated with NVCF, based on various aspects of patient information, including baseline data, laboratory examinations, imaging findings, and surgical records. Baseline characteristics included age at the time of injury, age at menopause, body mass index (BMI), bone mineral density (BMD) [29], history of hypertension, history of diabetes, time elapsed between injury and operation, duration of hospital stay, location of fracture, presence of multiple vertebral fractures, history of steroid use, and regular anti-osteoporosis treatment after the initial operation. Imaging examination variables included the preoperative local kyphosis Cobb angle of the initial fracture, postoperative local kyphosis Cobb angle, change in the local Cobb angle, vertebral body recovery height, vertebral height recovery rate, presence of endplate fracture (defined as discontinuous fracture lines in the endplate on computed tomography (CT) images), bone cement leakage (defined as leakage extending beyond the upper or lower vertebral lamina into the intervertebral space on radiographic or CT images), and uniformity of bone cement diffusion (indicating excellent diffusion when the cement exhibited a uniform distribution on both sides of the vertebral body midline and formed a close adhesion to the upper and lower endplates). Laboratory indicators included osteocalcin, vitamin D, and parathyroid hormone levels [30, 31]. Surgical factors included the amount of bone cement used and number of vertebral bodies involved.

### Statistical analysis

All statistical analyses were conducted using STATA 17.0 for Windows (StataCorp, Texas, USA) and R version 4.1.3 (R Foundation for Statistical Computing, Vienna, Austria). Continuous variables were assessed using the Mann–Whitney *U* test, while categorical variables were assessed using the Chi-square test or Fisher’s exact test. Continuous and categorical variables are presented as medians (quartiles) and the number of cases (percentage), respectively.

A computer was used for random sampling in a fixed proportion, with patients assigned to the training set (70%) and the verification set (30%). The LASSO regression method was employed to reduce data dimensionality, prevent overfitting and multicollinearity, and identify characteristic variables associated with NVCF. A tenfold cross-validation was conducted to determine the optimal λ value based on 1 standard error. A predictive model was established using a combination of multifactor logistic regression analyses. The training set was used to develop the nomogram and calibration curves using R software and the "rms" software package. The receiver operating characteristic (ROC) curve was plotted using Stata software to assess the discriminative ability of the model, where a larger area under the ROC curve (AUC) indicated stronger performance. A decision curve was generated using Stata software to quantitatively verify the net benefit across different threshold probabilities and assess the clinical applicability of the model. *P* values < 0.05 were considered statistically significant.

## Results

### Patient baseline characteristics

In total, 357 patients met the inclusion criteria, consisting of 94 patients in the NVCF group and 263 patients in the non-NVCF group. No significant differences in the following characteristics were found between the two groups: age at the time of injury, age at menopause, history of hypertension, diabetes, and steroid use, BMI, BMD, multiple vertebral fractures, fracture site, time elapsed between injury and operation, duration of hospital stay, number of operative vertebrae, amount of bone cement injected, difference in vertebral height before and after the operation, recovery rate of vertebral height, preoperative and postoperative local Cobb angles, recovery of Cobb angle, vitamin D level, and parathyroid hormone level (all *P* > 0.05). However, we found that the NCVF group received less anti-osteoporosis treatment than the non-NCVF group (*P* < 0.05) and a significant difference in the osteocalcin levels between the two groups (*P* < 0.01). The NCVF group exhibited a higher prevalence of preoperative vertebral endplate fractures, intraoperative bone cement leakage, and poor cement dispersion (*P* < 0.01). Table 1 presents the general characteristics of patients in the model population, including the training and testing sets.

**Table 1:** Baseline characteristics of patients in the Non-NVCF and NVCF groups

Variables	Non-NVCF group	NVCF group	*P* value
	(*n* = 263)	(*n* = 94)	
Age (years)			0.31
≤ 70	123 (46.8%)	36 (38.3%)	
80 > age > 70	92 (35.0%)	36 (38.3%)	
≥ 80	48 (18.3%)	22 (23.4%)	
Menopausal age			0.73
≤ 47	67 (25.5%)	20 (21.3%)	
52 > age > 47	160 (60.8%)	60 (63.8%)	
≥ 53	36 (13.7%)	14 (14.9%)	
History of hypertension			0.46
No	164 (62.4%)	54 (57.4%)	
Yes	99 (37.6%)	40 (42.6%)	
History of diabetes			0.52
No	221 (84.0%)	76 (80.9%)	
Yes	42 (16.0%)	18 (19.1%)	
BMI (kg/m^2^), (median, IQR)	23.4 (21.5, 25.2)	23.3(20.7,26.4)	0.75
BMD (median, IQR)	0.6 (0.6, 0.7)	0.6 (0.6, 0.7)	0.21
Multiple vertebral fracture			0.12
No	207 (78.7%)	66 (70.2%)	
Yes	56 (21.3%)	28 (29.8%)	
Fracture site			0.11
Over T10	17 (6.5%)	8 (8.5%)	
T10–L2	176 (66.9%)	58 (61.7%)	
L3–L5	40 (15.2%)	9 (9.6%)	
Other	30 (11.4%)	19 (20.2%)	
Steroid use			1.00
No	252 (95.8%)	91 (96.8%)	
Yes	11 (4.2%)	3 (3.2%)	
Anti-osteoporosis therapy			0.04
No	113 (43.0%)	52 (55.3%)	
Yes	150 (57.0%)	42 (44.7%)	
Cement leakage			< 0.01
No	243 (92.4%)	14 (14.9%)	
Yes	20 (7.6%)	80 (85.1%)	
Bone cement diffusion			< 0.01
No	16 (9.1%)	78 (83.0%)	
Yes	247 (93.9%)	16 (17.0%)	
Endplate fracture			< 0.01
No	252 (95.8%)	10 (10.6%)	
Yes	11 (4.2%)	84 (89.4%)	
Fractured vertebral bodies (numbers)	1.0 (1.0, 1.0)	1.0 (1.0, 2.0)	0.09
Hospitalization to surgery (days)	3.0 (2.0, 4.0)	3.0 (2.0, 5.0)	0.40
Operation time after injury (days)	9.0 (4.0, 18.0)	10.0 (6.0, 18.0)	0.43
Osteocalcin	19.0 (14.6, 26.1)	16.3 (12.9, 23.5)	< 0.01
Vitamin D	15.5 (11.5, 21.4)	16.2 (11.2, 22.9)	0.73
Injection volume (mL)	5.0 (4.0, 5.0)	4.5 (3.5, 5.0)	0.14
Parathyroid hormone	41.2 (30.7 53.0)	39.4 (32.3, 52.7)	0.91
Height recovery (mm)	9.4 (6.8,12.1)	9.1 (6.5,12.0)	0.51
High recovery rate	0.7 (0.4, 0.9)	0.7 (0.5, 1.0)	0.40
Preoperative Cobb angle (°)	14.1 (10.7, 18.2)	13.7 (11.1, 19.7)	0.41
Postoperative Cobb angle (°)	4.0 (2.0,6.2)	3.9 (1.8,6.9)	0.69
Cobb angle recovery (°)	9.9 (7.3,13.4)	11.0 (6.9,14.9)	0.35

NVCF, new vertebral compression fractures; BMI, body mass index; BMD, bone mass density; IQR, interquartile range

### LASSO regression analysis results

**Fig. 1:**
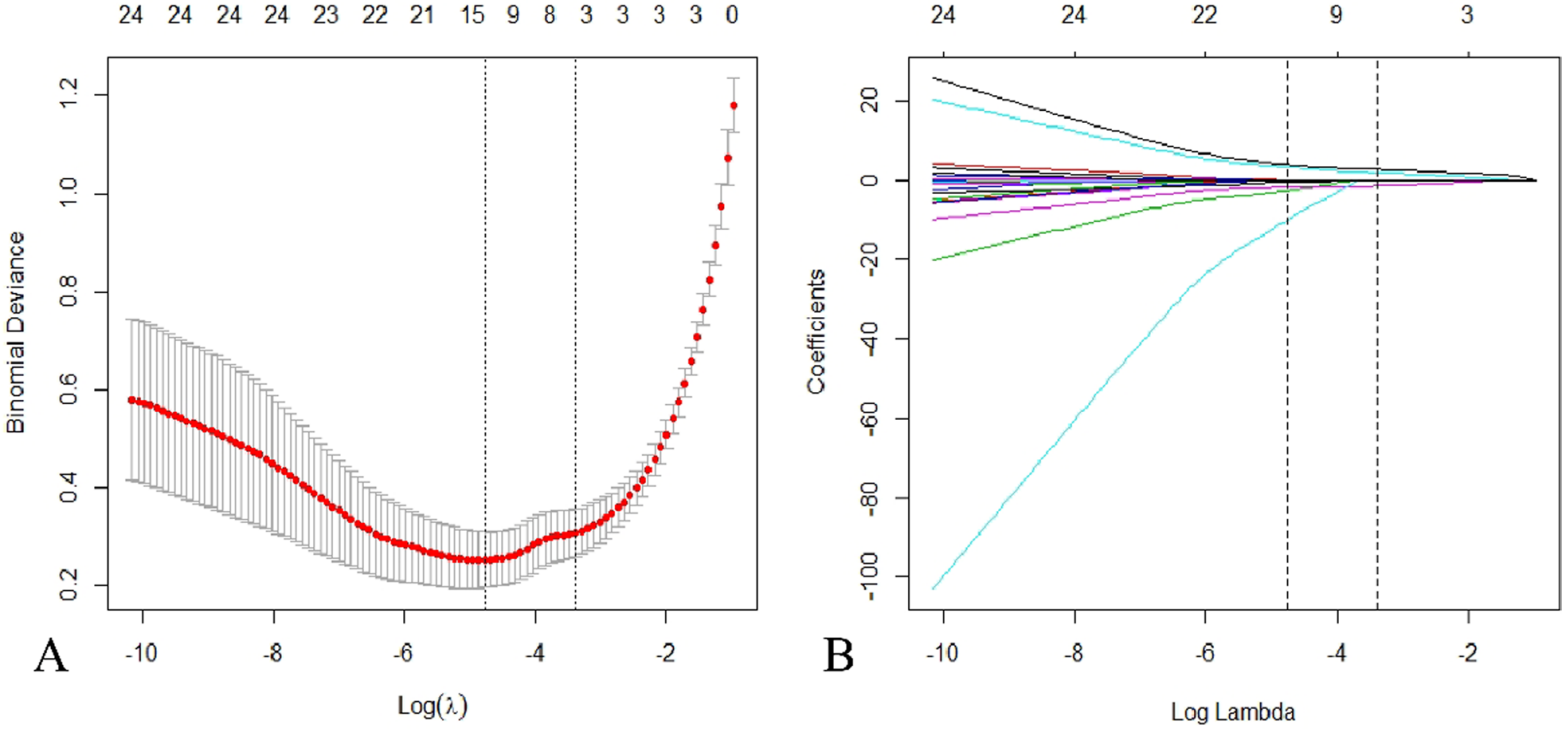
LASSO regression analysis. **A** Determining the optimal lambda value for LASSO regression using cross-validation. The model achieves the best performance when the mean square error of distance is twice the standard error (*λ* 1se). **B** Variations in LASSO regression coefficients are shown

LASSO regression analysis was employed to screen the characteristic variables and generate variable shrinkage coefficient maps and cross-validation curves. The optimal model was obtained when the mean square error of distance was twice the standard error (*λ* 1se). Three research variables with nonzero coefficients were selected: bone cement leakage, uneven dispersion of bone cement, and fracture destruction of the endplate (Fig. 1).

### Multivariable logistic regression analysis

A model was established, with bone cement leakage, poor cement dispersion, and endplate fracture as independent variables and new fracture as the dependent variable. Multivariate logistic regression analysis revealed a significant association between bone cement leakage, poor cement dispersion, endplate fractures, and new fractures (*P* < 0.01). The detailed results are presented in Table 2.

**Table 2:** Prediction factors for NVCF

Intercept and variable	Prediction model			
	Odds ratio [95% CI]	Std. err	*Z*	*P* >|*Z*|
Cement leakage	22.72399 [5.3446287,96.5866]	16.78	4.23	< 0.001
Bone cement diffusion	0.1108337 [0.0278436,0.4411833]	0.08	− 3.12	0.002
Endplate fracture	52.63849 [12.51557,221.3891]	38.58	5.41	< 0.001
Cons	0.0647761 [0.0131643,0.3187363]	0.05	− 3.37	0.001

CI, confidence interval, Std. Err, standard error; Cons: estimates baseline odds

### Development and verification of the nomogram

**Fig. 2:**
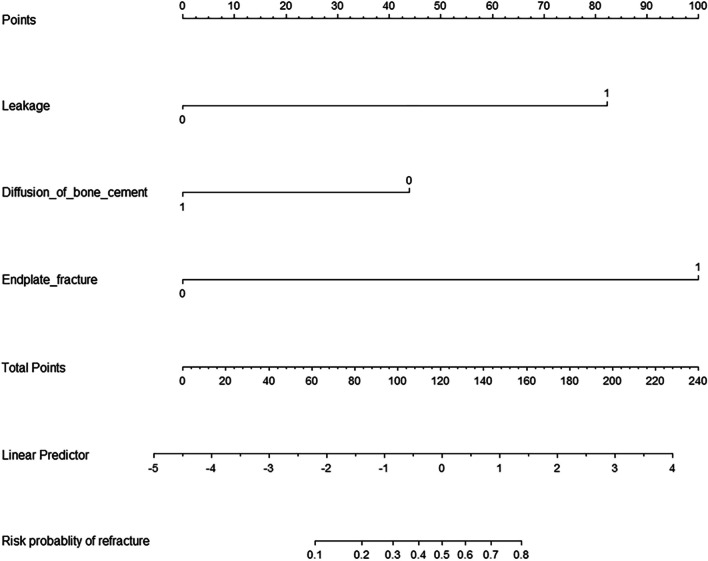
Nomogram for predicting NVCF risk after PKP in postmenopausal women

**Fig. 3:**
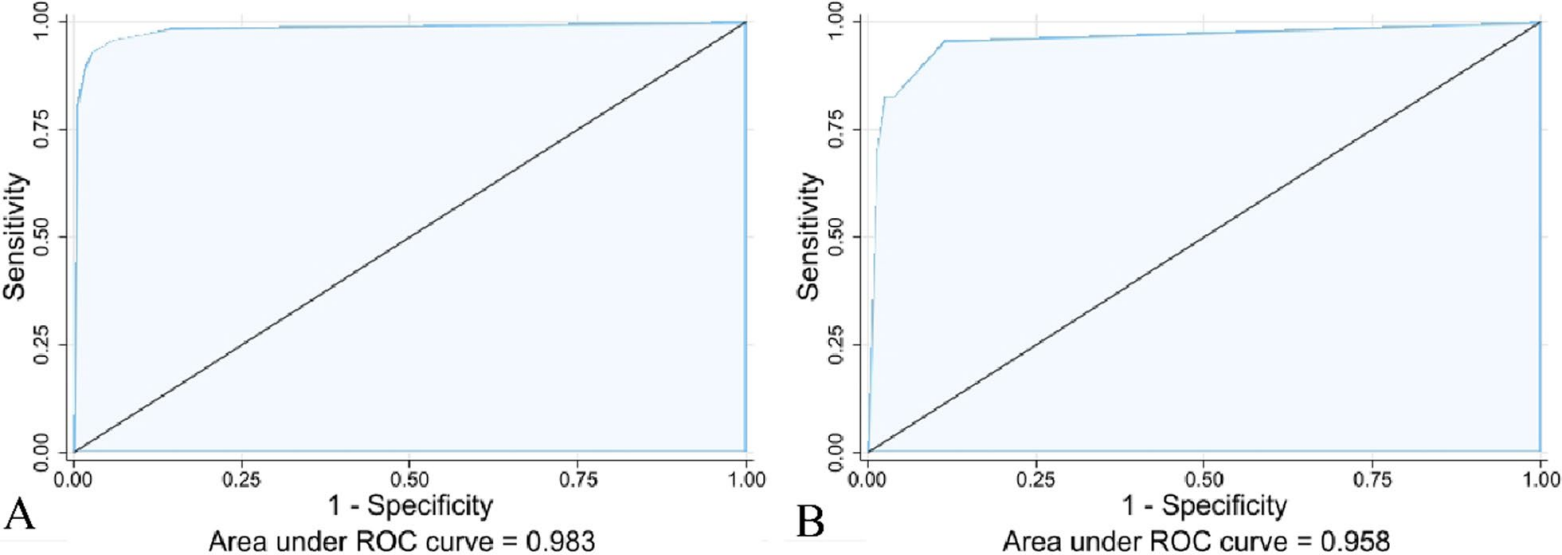
Receiver operating characteristic (ROC) curve. The ROC curve represents the relationship between sensitivity and specificity, with the area under the curve (AUC) serving as an indicator of prediction accuracy. The training (**A**) and testing (**B**) sets had an AUC of 0.983 and 0.958, respectively

**Fig. 4:**
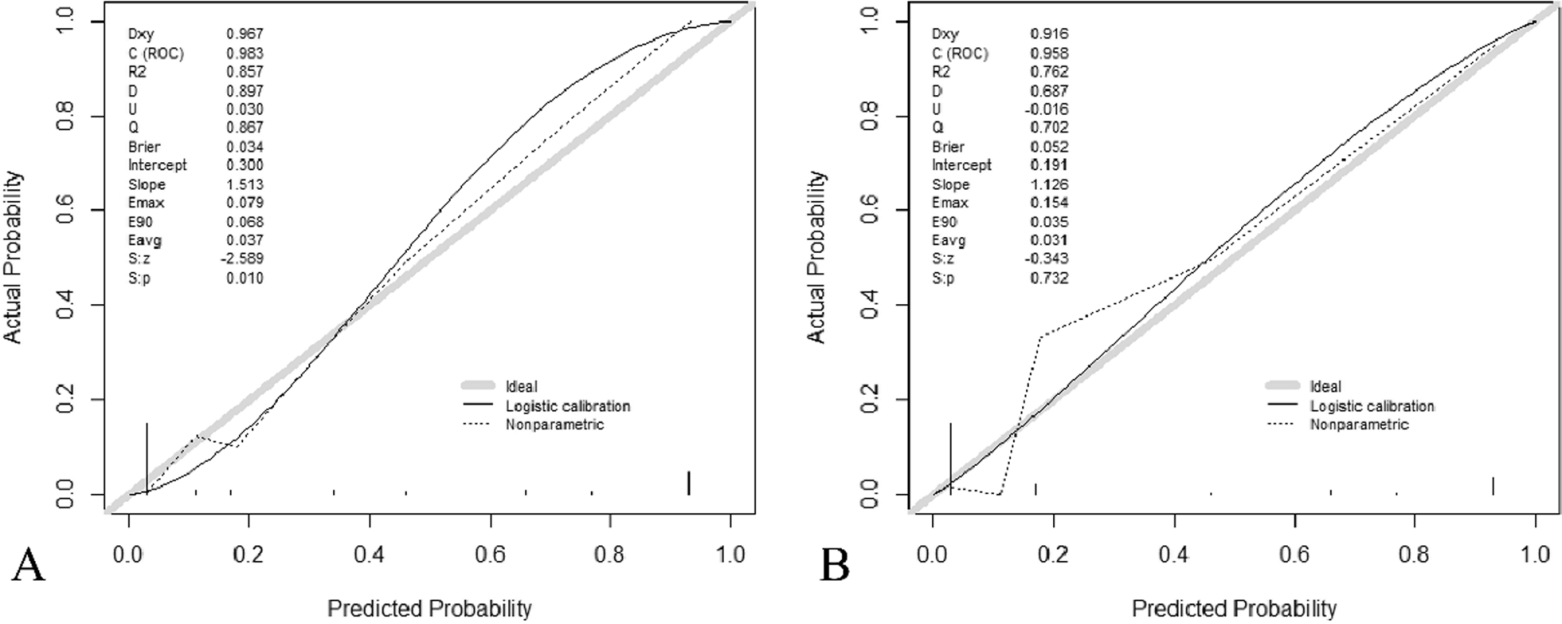
Calibration curves. The diagonal gray solid line represents the model’s ideal prediction. The solid black line indicates the performance of the line chart, with closer proximity to the diagonal indicating more accurate predictions within the training (**A**) and testing (**B**) sets

**Fig. 5:**
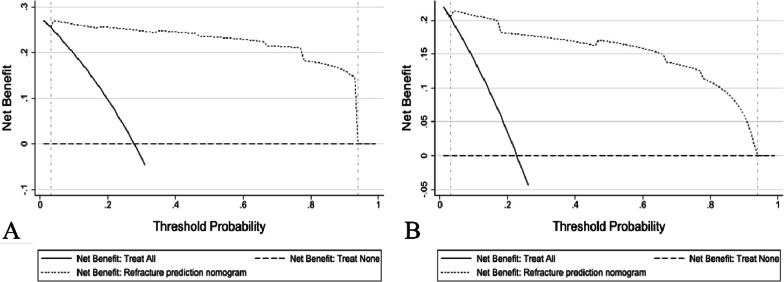
Decision curve analysis. The *y*-axis represents net benefit, which is the highest within the threshold range of approximately 0.03–0.94 among the training (**A**) and testing (**B**) sets

The nomogram was constructed based on the three independent predictors: bone cement leakage, poor dispersion of bone cement, and endplate fractures. Each predictor was assigned a score, and the total score was calculated by summating the individual scores. A straight line was drawn to predict the probability of refracture (Fig. 2). ROC curve analysis revealed an AUC of 0.983 for the training set and 0.958 for the verification set, indicating the model's strong predictive ability (Fig. 3). Moreover, the calibration curve showed a good fit with minimal deviation, demonstrating the model's high discriminant ability and excellent fit (Fig. 4). DCA revealed that the net benefit of the predictive model was significantly higher than both the intervention and non-intervention outcomes for all patients when the risk threshold ranged between 0.03 and 0.94 (Fig. 5).

## Discussion

Osteoporosis poses a significant threat to the health of postmenopausal women, particularly due to its primary complication, OVCF, which is associated with high morbidity and mortality rates in this population [1, 32]. Given the high incidence of NVCF after PKP in postmenopausal women, we constructed a nomogram based on three independent predictors identified using LASSO regression: cement leakage, cement dispersion, and endplate fracture. The nomogram developed in this study is capable of predicting postoperative NVCF with high differentiation accuracy and broad clinical applicability in postmenopausal women [33, 34]. Notably, our results differed from those of previously published studies that analyzed the risk factors associated with NVCF after PKP. These disparities can be attributed to variations in surgical practices, surgical expertise, and healthcare standards across different countries.

Polymethyl methacrylate is a common material used in bone cement [35]. When injected into the vertebral body, it can alleviate pain by desensitizing nerve endings and stabilizing vertebral structures. However, bone cement is prone to leakage during PKP and leakage detection is challenging [36]. Churojana et al. [37] found that cement leakage into the intervertebral disc did not increase the risk of NVCF. In contrast, a retrospective analysis conducted by Choi et al. [38] reported a strong correlation between bone cement leakage and recurrent vertebral fractures at 1-year follow-up, consistent with the results of our study. The intervertebral disc serves as a crucial biomechanical buffer for the spinal cord. The infiltration of bone cement into the intervertebral disc reduces its buffering capacity, thereby increasing the incidence of spinal NVCF [39]. Consistent with our findings, a long-term follow-up study conducted by Chen et al. [40] revealed a statistically significant difference in intervertebral disc leakage in the group with adjacent vertebral fractures. Generally, bone cement increases mechanical load transmission between adjacent vertebrae [41]. This elevated stress further increases the risk of NVCF, particularly considering the rigid nature of the cement material within the intervertebral disc. Researchers have described NVCF resulting from these strength differences as a direct column effect, whereas distal fractures are attributed to a dynamic hammer effect after bone cement leakage [42].

This study revealed that endplate integrity disruption was a predictor of NVCF. The vertebral endplate functions as an intermediate structure that connects the vertebral body to the intervertebral disc and transfers the mechanical load within the spine. According to Ortiz et al. [43], endplate injuries influence the prognosis of patients with OVCF by causing uneven axial loading and an increased risk of refracture [44]. Several studies have suggested that endplate destruction leads to stress concentration, thereby compromising the biomechanical stability of the spinal column. Moreover, the loss of endplate integrity may lead to bone cement leakage, both of which contribute to the increased incidence of NVCF [45, 46]. However, further investigations are required to determine whether endplate repair can mitigate NVCF occurrence in patients with osteoporosis. Based on our findings, a thorough preoperative examination of imaging data, the appropriate adjustment of bone cement viscosity [47], and close monitoring the working channel’s interaction with the endplate are necessary for NVCF prevention.

In this study, imaging data analysis demonstrated that the bone cement crossed the midline of the vertebral body and closely fitted the upper and lower endplates with good dispersion. Moreover, multivariate logistic regression analysis revealed a statistically significant difference in bone cement diffusion between the NVCF and non-NVCF groups. In a retrospective analysis [48] of 217 patients with single vertebral fractures, the refracture rate in the group with bone cement diffusion across the midline (6.7%) was significantly lower than that of the group with unilateral diffusion (15.7%), consistent with our findings. The uneven distribution of bone cement, especially its asymmetrical distribution in the coronal plane, leads to a substantial difference in stiffness between the two sides, resulting in uneven stress distribution [49, 50]. This imbalance increases the risk of biomechanical abnormalities contributing to NVCF. Song et al. proposed that uniformly distributed bone cement not only increases the strength of the affected vertebrae but also better withstands body rotation-related stress, thereby reducing the risk of new fractures in the adjacent vertebrae. Additionally, studies have shown that when bone cement is in close contact with the endplate and forms a ring near the endplate, it can prevent an overall biomechanical stress imbalance and reduce the risk of NVCF [51]. Therefore, we recommend that bone cement be evenly and symmetrically distributed during the PKP process, maximizing contact with the upper and lower plates, increasing the contact area between the implant material and the bone structure, and ensuring the stability of the bone cement [52] to avoid cement leakage.

We established a nomogram to predict the risk of developing NVCF after PKP in postmenopausal women. Nomograms have been previously validated for their effectiveness and reliability in various disease models [53, 54]. By combining literature reports, clinical experience, imaging data, and a large sample, we employed LASSO regression to select a reduced number of predictive factors for inclusion in the model. The constructed nomogram was evaluated for differentiation, calibration, and clinical applicability. This model is a valuable tool for spinal surgeons as it can predict the likelihood of NVCF in postmenopausal women, potentially extending the life expectancy of this high-risk group [52]. Additionally, it reminds clinicians to extend the follow-up period, reduce re-examination intervals, and inform postmenopausal female patients about the potential risks. Moreover, utilizing this model can significantly mitigate the unnecessary consumption of medical resources.

This study had several limitations. First, all cases were derived from orthopedic in-patients at our hospital; hence, there is a lack of multicenter comparisons with large sample data. Therefore, further external validation, using patient data from diverse countries and regions, is required. Second, the retrospective design of this study introduces varying degrees of selection bias. Therefore, it is crucial to conduct prospective studies in collaboration with other research institutions to verify the accuracy and generalizability of the nomogram.

## Conclusions

Using multivariate logistic and LASSO regression analyses, cement leakage, uneven cement dispersion, and endplate fracture were identified as independent predictors of postoperative NVCF in postmenopausal women. Based on these factors, we established a nomogram capable of predicting postoperative NVCF with high differentiation accuracy and broad clinical applicability. This nomogram is a valuable tool that can aid clinicians in evaluating the prognostic risk and guide clinical decision-making for postmenopausal women to mitigate NVCF.

## Data Availability

The datasets used and/or analyzed in the study are available from the corresponding author upon reasonable request.

## Abbreviations

NVCFs: New vertebral compression fractures
OVCFs: Osteoporotic vertebral compression fractures
PKP: Percutaneous kyphoplasty
ROC: Receiver operating characteristic
DCA: Decision curve analysis
OP: Osteoporosis
MRI: Magnetic resonance imaging
BMI: Body mass index
BMD: Bone mineral density
AUC: Area under the ROC curve
PMMA: Polymethyl methacrylate

## References

[1] The North American Menopause Society. Management of osteoporosis in postmenopausal women: the 2021 position statement of The North American Menopause Society. Menopause. 2021;28(9):973–97. 10.1097/GME.0000000000001831 34448749

[2] Migliorini F, Colarossi G, Eschweiler J (2022). Antiresorptive treatments for corticosteroid-induced osteoporosis: a Bayesian network meta-analysis. Br Med Bull.

[3] NIH Consensus Development Panel on Osteoporosis Prevention, Diagnosis, and Therapy. Osteoporosis prevention, diagnosis, and therapy. JAMA. 2001;285(6):785–95. 10.1001/jama.285.6.785 11176917

[4] Finkelstein JS, Brockwell SE, Mehta V (2008). Bone mineral density changes during the menopause transition in a multiethnic cohort of women. J Clin Endocrinol Metab.

[5] Farr JN, Khosla S (2015). Skeletal changes through the lifespan—from growth to senescence. Nat Rev Endocrinol.

[6] Migliorini F, Colarossi G, Baroncini A (2021). Pharmacological management of postmenopausal osteoporosis: a level I evidence based-expert opinion. Expert Rev Clin Pharmacol.

[7] Buchbinder R, Johnston RV, Rischin KJ (2018). Percutaneous vertebroplasty for osteoporotic vertebral compression fracture. Cochrane Database Syst Rev.

[8] Kendler DL, Bauer DC, Davison KS (2016). Vertebral fractures: clinical importance and management. Am J Med.

[9] Hernlund E, Svedbom A, Ivergård M (2013). Osteoporosis in the European Union: medical management, epidemiology and economic burden. A report prepared in collaboration with the International Osteoporosis Foundation (IOF) and the European Federation of Pharmaceutical Industry Associations (EFPIA). Arch Osteoporos.

[10] Johnell O, Kanis JA (2006). An estimate of the worldwide prevalence and disability associated with osteoporotic fractures. Osteoporos Int.

[11] Bow CH, Cheung E, Cheung CL (2012). Ethnic difference of clinical vertebral fracture risk. Osteoporos Int.

[12] Kaliya-Perumal AK, Lin TY (2018). Clinical outcomes of percutaneous vertebroplasty for selective single segment dorsolumbar vertebral compression fractures. J Clin Orthop Trauma.

[13] Wang F, Wang LF, Miao DC (2018). Which one is more effective for the treatment of very severe osteoporotic vertebral compression fractures: PVP or PKP?. J Pain Res.

[14] Wang LJ, Yang HL, Shi YX (2012). Pulmonary cement embolism associated with percutaneous vertebroplasty or kyphoplasty: a systematic review. Orthop Surg.

[15] Sun HB, Shan JL, Tang H (2021). Percutaneous vertebral augmentation for osteoporotic vertebral compression fractures will increase the number of subsequent fractures at adjacent vertebral levels: a systematic review and meta-analysis. Eur Rev Med Pharmacol Sci.

[16] Lee BG, Choi JH, Kim DY (2019). Risk factors for newly developed osteoporotic vertebral compression fractures following treatment for osteoporotic vertebral compression fractures. Spine J.

[17] Movrin I, Vengust R, Komadina R (2010). Adjacent vertebral fractures after percutaneous vertebral augmentation of osteoporotic vertebral compression fracture: a comparison of balloon kyphoplasty and vertebroplasty. Arch Orthop Trauma Surg.

[18] Lindsay R, Silverman SL, Cooper C (2001). Risk of new vertebral fracture in the year following a fracture. JAMA.

[19] Nieuwenhuijse MJ, Putter H, van Erkel AR (2013). New vertebral fractures after percutaneous vertebroplasty for painful osteoporotic vertebral compression fractures: a clustered analysis and the relevance of intradiskal cement leakage. Radiology.

[20] Kanis JA, Johansson H, Odén A (2018). Characteristics of recurrent fractures. Osteoporos Int.

[21] Li Q, Long X, Wang Y (2021). Development and validation of a nomogram for predicting the probability of new vertebral compression fractures after vertebral augmentation of osteoporotic vertebral compression fractures. BMC Musculoskelet Disord.

[22] Park JS, Park YS (2021). Survival analysis and risk factors of new vertebral fracture after vertebroplasty for osteoporotic vertebral compression fracture. Spine J.

[23] Bian F, Bian G, Zhao L (2022). Risk factors for recollapse of new vertebral compression fractures after percutaneous kyphoplasty in geriatric patients: establishment of a nomogram. BMC Musculoskelet Disord.

[24] Si F, Yuan S, Zang L (2022). Paraspinal muscle degeneration: a potential risk factor for new vertebral compression fractures after percutaneous kyphoplasty. Clin Interv Aging.

[25] Gao W, Chen Y, Wang X (2023). Establishment and verification of a predictive nomogram for new vertebral compression fracture occurring after bone cement injection in middle-aged and elderly patients with vertebral compression Fracture. Orthop Surg.

[26] Zhang ZL, Yang JS, Hao DJ (2021). Risk factors for new vertebral fracture after percutaneous vertebroplasty for osteoporotic vertebral compression fractures. Clin Interv Aging.

[27] Wang H, Chen X, Zhao J (2020). Predictive nomogram for midterm to long-term prognosis in patients with papillary renal cell carcinoma based on data from the surveillance, epidemiology, and end results (SEER) program. Med Sci Monit.

[28] Park SY (2018). Nomogram: an analogue tool to deliver digital knowledge. J Thorac Cardiovasc Surg.

[29] Conti V, Russomanno G, Corbi G (2015). A polymorphism at the translation start site of the vitamin D receptor gene is associated with the response to anti-osteoporotic therapy in postmenopausal women from southern Italy. Int J Mol Sci.

[30] Migliorini F, Maffulli N, Spiezia F (2021). Potential of biomarkers during pharmacological therapy setting for postmenopausal osteoporosis: a systematic review. J Orthop Surg Res.

[31] Migliorini F, Maffulli N, Spiezia F (2021). Biomarkers as therapy monitoring for postmenopausal osteoporosis: a systematic review. J Orthop Surg Res.

[32] Migliorini F, Maffulli N, Colarossi G (2021). Effect of drugs on bone mineral density in postmenopausal osteoporosis: a Bayesian network meta-analysis. J Orthop Surg Res.

[33] Karakousis G, Sondak VK, Zager JS (2020). Overestimation of risk for sentinel lymph node metastasis in a nomogram for T1 melanomas. J Clin Oncol.

[34] Kao WY, Su CW, Chiou YY (2017). Hepatocellular carcinoma: nomograms based on the albumin-bilirubin grade to assess the outcomes of radiofrequency ablation. Radiology.

[35] Lai PL, Chen LH, Chen WJ (2013). Chemical and physical properties of bone cement for vertebroplasty. Biomed J.

[36] Martikos K, Greggi T, Faldini C (2018). Osteoporotic thoracolumbar compression fractures: long-term retrospective comparison between vertebroplasty and conservative treatment. Eur Spine J.

[37] Churojana A, Songsaeng D, Khumtong R (2014). Is intervertebral cement leakage a risk factor for new adjacent vertebral collapse?. Interv Neuroradiol.

[38] Choi SS, Kim H, Choung YJ (2022). Risk factors for new vertebral compression fracture after kyphoplasty and efficacy of osteoporosis treatment: a STROBE-compliant retrospective study. Medicine (Baltimore).

[39] Sun YC, Teng MM, Yuan WS (2011). Risk of post-vertebroplasty fracture in adjacent vertebral bodies appears correlated with the morphologic extent of bone cement. J Chin Med Assoc.

[40] Chen WJ, Kao YH, Yang SC (2010). Impact of cement leakage into disks on the development of adjacent vertebral compression fractures. J Spinal Disord Tech.

[41] Belkoff SM, Mathis JM, Erbe EM (2000). Biomechanical evaluation of a new bone cement for use in vertebroplasty. Spine (Phila Pa 1976).

[42] Ahn Y, Lee JH, Lee HY (2008). Predictive factors for subsequent vertebral fracture after percutaneous vertebroplasty. J Neurosurg Spine.

[43] Ortiz AO, Bordia R (2011). Injury to the vertebral endplate-disk complex associated with osteoporotic vertebral compression fractures. AJNR Am J Neuroradiol.

[44] Uppin AA, Hirsch JA, Centenera LV (2003). Occurrence of new vertebral body fracture after percutaneous vertebroplasty in patients with osteoporosis. Radiology.

[45] Su Y, Ren D, Chen Y (2023). Effect of endplate reduction on endplate healing morphology and intervertebral disc degeneration in patients with thoracolumbar vertebral fracture. Eur Spine J.

[46] Zhao Z, Deng L, Hua X (2022). A retrospective study on the efficacy and safety of bone cement in the treatment of endplate fractures. Front Surg.

[47] Lu Q, Gao S, Zhou M (2021). The effect of bone cement on the curative effect of percutaneous kyphoplasty in the treatment of osteoporotic vertebral compression fracture. Ann Palliat Med.

[48] Song Q, Zhao Y, Li D (2023). Effect of different bone cement distributions in percutaneous kyphoplasty on clinical outcomes for osteoporotic vertebral compression fractures: a retrospective study. Medicine (Baltimore).

[49] Kim MJ, Lindsey DP, Hannibal M (2006). Vertebroplasty versus kyphoplasty: biomechanical behavior under repetitive loading conditions. Spine (Phila Pa 1976).

[50] Zhao Y, Robson Brown KA, Jin ZM (2012). Trabecular level analysis of bone cement augmentation: a comparative experimental and finite element study. Ann Biomed Eng.

[51] Baek SW, Kim C, Chang H (2015). The relationship between the spinopelvic balance and the incidence of adjacent vertebral fractures following percutaneous vertebroplasty. Osteoporos Int.

[52] Migliorini F, Giorgino R, Hildebrand F (2021). Fragility fractures: risk factors and management in the elderly. Medicina (Kaunas).

[53] Cai J, Cheng J, Li H (2019). A nomogram for the prediction of cerebrovascular disease among patients with brain necrosis after radiotherapy for nasopharyngeal carcinoma. Radiother Oncol.

[54] Hoshino N, Hida K, Sakai Y (2018). Nomogram for predicting anastomotic leakage after low anterior resection for rectal cancer. Int J Colorectal Dis.

